# *Melissa officinalis* Extract Inhibits Laser-Induced Choroidal Neovascularization in a Rat Model

**DOI:** 10.1371/journal.pone.0110109

**Published:** 2014-10-14

**Authors:** Eun Kyoung Lee, Young Joo Kim, Jin Young Kim, Hyun Beom Song, Hyeong Gon Yu

**Affiliations:** 1 Department of Ophthalmology, Seoul National University College of Medicine, Seoul National University Hospital, Seoul, Korea; 2 Department of Ophthalmology, Seoul National University Hospital Biomedical Research Institute, Seoul, Korea; 3 Department of Biomedical Sciences, Seoul National University Graduate School, Seoul, Korea; Medical University of South Carolina, United States of America

## Abstract

**Purpose:**

This study investigated the effect of *Melissa officinalis* extract on laser-induced choroidal neovascularization (CNV) in a rat model. The mechanism by which *M. officinalis* extract acted was also investigated.

**Methods:**

Experimental CNV was induced by laser photocoagulation in Brown Norway rats. An active fraction of the *Melissa* leaf extract was orally administered (50 or 100 mg/kg/day) beginning 3 days before laser photocoagulation and ending 14 days after laser photocoagulation. Optical coherence tomography and fluorescein angiography were performed *in vivo* to evaluate the thickness and leakage of CNV. Choroidal flat mount and histological analysis were conducted to observe the CNV *in vitro*. Vascular endothelial growth factor (VEGF), matrix metalloproteinase (MMP)-2, and MMP-9 expression were measured in retinal and choroidal-scleral lysates 7 days after laser injury. Moreover, the effect of *M. officinalis* extract on tertiary-butylhydroperoxide (t-BH)-induced VEGF secretion and mRNA levels of VEGF, MMP-2, and MMP-9 were evaluated in human retinal epithelial cells (ARPE-19) as well as in human umbilical vein endothelial cells (HUVECs).

**Results:**

The CNV thickness in *M. officinalis*-treated rats was significantly lower than in vehicle-treated rats by histological analysis. The CNV thickness was 33.93±7.64 µm in the high-dose group (*P*<0.001), 44.09±12.01 µm in the low-dose group (*P* = 0.016), and 51.00±12.37 µm in the control group. The proportion of CNV lesions with clinically significant fluorescein leakage was 9.2% in rats treated with high-dose *M. officinalis*, which was significantly lower than in control rats (53.4%, *P*<0.001). The levels of VEGF, MMP-2, and MMP-9 were significantly lower in the high-dose group than in the control group. Meanwhile, *M. officinalis* extract suppressed t-BH-induced transcription of VEGF and MMP-9 in ARPE-19 cells and HUVECs.

**Conclusions:**

Systemic administration of *M. officinalis* extract suppressed laser-induced CNV formation in rats. Inhibition of VEGF and MMP-9 via anti-oxidative activity may underlie this effect.

## Introduction

Neovascular diseases of the retina include age-related macular degeneration (AMD) and diabetic retinopathy, and together they comprise the leading causes of adult-onset blindness in developed countries [1]. In particular, exudative AMD is characterized by choroidal neovascularization (CNV), which grows through breaks in Bruch’s membrane into the subretinal or sub-retinal pigment epithelium (RPE) space [2], [3]. Although the pathogenesis of AMD is not fully understood, various clinical studies have shown that vascular endothelial growth factor (VEGF) plays a central role in CNV pathogenesis. The importance of VEGF in CNV development has led to the use of various anti-VEGF therapies. Among these therapeutic agents, the anti-VEGF antibody drugs, ranibizumab (Lucentis, Genentech, South San Francisco, CA), bevacizumab (Avastin, Genentech), and aflibercept (Eylea, Bayer HealthCare, Berlin, Germany) are most widely used to treat AMD in the clinical setting, and have similar efficacy and side effects [4]–[6].

The matrix metalloproteinase (MMP) family of enzymes also has important roles in CNV progression. Both MMP-2 and MMP-9 are of particular interest because of their substrate specificity to type IV collagen, which must degrade before vascular endothelial cell migration can occur [7]. Additionally, MMP-2 and MMP-9 are localized to Bruch’s membrane in areas of new vessel formation [8]–[10] and disruption of the *mmp-2*, *mmp-9*, or double *mmp-2/mmp-9* genes inhibits CNV complex formation, induced when Bruch’s membrane ruptures [7]. Moreover, administration or over expression of MMP-2 and MMP-9 enzymatic inhibitors blocks experimental CNV formation [11], [12]. In the eye, strong expression of MMP-2 and MMP-9 has been measured in choroidal neovascular membranes surgically removed from patients with AMD [8]. This suggests that MMP-2 and MMP-9 could contribute to CNV progression.

Recently, oxidative stress, caused by reactive oxygen species (ROS), has also been shown to be involved in the progression of CNV. Hara et al. [13] reported the up-regulation of 4-hydroxy-2-nonenal (HNE)-modified protein, an oxidative stress marker, and nuclear factor (NF)-κB, a redox-sensitive transcription factor, in a laser-induced experimental CNV model. They also showed that *N*-acetyl-cysteine (NAC), an antioxidant and a free radical-scavenging agent which increases intracellular glutathione (GSH) levels, clearly suppressed the activation of NF-κB, after suppressing the oxidative stress induced by laser injury [13]. Roggia et al. [14] also demonstrated that glutathione peroxidase 4 (GPx4), an endogenous antioxidant enzyme, suppresses the increase in the VEGF-A protein level and confers protection against laser-induced CNV development *in vivo*. Based on these results, oxidative stress has been implicated in the development of laser-induced CNV.

Although current therapeutic options to treat CNV are effective in the majority of patients, these drugs are intravitreally administered. Frequent intravitreal injections may increase procedure-related side effects, including endophthalmitis and retinal detachment [15]–[18]. Therefore, many researchers are trying to develop new therapies that have improved efficacy, reduced cost, easier administration, and fewer associated complications. *Melissa officinalis* L. (Labiatae; lemon balm) extract is a traditional phytomedicine that is widely used as a mild sedative, spasmolytic, antiviral, and antibacterial agent [19]–[21]. *Melissa* was also recently shown to reduce adipose tissue mass in obese mice through reduction of mRNA levels of angiogenic factors (VEGF-A and fibroblast growth factor-2) and MMPs (MMP-2 and MMP-9) [22], [23].

In the present study, we investigate whether an active fraction of *Melissa* leaf extract can limit CNV formation. Molecular mechanisms underlying its effects on laser-induced CNV were also evaluated.

## Materials and Methods

### *Melissa officinalis* Leaf Extraction and Isolation

The *M. officinalis* extract (ALS-L1023; AngioLab, Inc., Daejeon, Korea) was manufactured from the leaves of *Melissa officinalis* L. (Alfred Galke GmbH, Harz, Germany) using activity-guided fractionation. The dried *Melissa* leaves were extracted with aqueous ethanol, and the extract was filtered and concentrated. The concentrated ethanol extract was further fractionated with ethyl acetate, after which it was further concentrated and dried to obtain *M. officinalis* extract in powder form. The *M. officinalis* extract was standardized using two reference compounds, rosmarinic acid and caffeic acid, by high performance liquid chromatography (HPLC) and it was dissolved in 100% dimethyl sulfoxide (DMSO) for use in *in vitro* tests.

### Animals

Seven week old male Brown Norway rats (Japan SLC, Hamamatsu, Japan), weighing 160–180 g, were used in this study. All procedures were approved by the Institutional Animal Care and Use Committee of the Seoul National University Hospital (Permit Number: 13-0068) and adhered to the Association for Research in Vision and Ophthalmology statement for the Use of Animals in Ophthalmic and Vision Research. During all procedures, including examination and photography, rats were anesthetized with a 1∶1 ketamine hydrochloride (Phoenix Pharmaceutical, St. Joseph, MO): xylazine hydrochloride (Phoenix Pharmaceutical) mixture (1 mL/kg) that was administered intramuscularly and all efforts were made to minimize suffering. Pupils were dilated with tropicamide (0.5% Mydrin M, Santen Pharmaceutical, Osaka, Japan).

### Administration of *M. officinalis* Extract and Control Vehicle

The *M. officinalis* extract was reconstituted by dissolving it in 100% DMSO and then suspending the resulting solution in a 0.5% carboxymethylcellulose (CMC) suspension (DMSO:CMC ratio = 1∶7) immediately before use. Animals were divided into three groups (16 rats per group), according to *M. officinalis* extract dose, which included vehicle only (control), 50 mg/kg/d (low-dose), and 100 mg/kg/d (high-dose). Rats in all groups were administered their assigned treatments orally. Three days before laser photocoagulation, all rats began receiving either drug or vehicle once a day. Drug and vehicle administration continued for 14 days after laser photocoagulation. After 18 days of *M. officinalis* extract treatment, no evidence of systemic adverse effects was observed in any study group.

### Laser-Induced Choroidal Neovascularization

After rats were anesthetized and pupils were dilated, rats were positioned on a Mayo stand in front of a laser-delivery system. The fundus was visualized using a microscope cover slip with 0.3% hydroxypropyl-methylcellulose (Genteal, Novartis Ophthalmics, Duluth, GA) as an optical coupling agent. A diode laser (Supra 577.Y, Quantel Medical SAS, Clermont-Ferrand, France) was used for photocoagulation (577 nm wavelength, 0.05 seconds duration, 100 µm spot size, 150 mW power). Ten to twelve lesions were created approximately 2–3 disc diameters from the optic nerve head. Bubble formation at the time of laser delivery indicated rupture of Bruch’s membrane and creation of a sufficient injury to induce CNV. Spots with hemorrhagic complications were excluded from further evaluation.

### Optical Coherence Tomography

Optical coherence tomography (OCT) was performed on anesthetized animals, using a spectral domain (SD)-OCT system (Cirrus OCT, Carl Zeiss Meditec, Dublin, CA), 14 days following laser photocoagulation. The CNV was represented on OCT as a spindle-shaped, subretinal, hyper-reflective material above the RPE layer. The horizontal and vertical cross-sectional images of the CNV lesion were obtained using a 5-line raster scan mode with length of 6 mm. The greatest linear dimension of the CNV lesion was measured using Cirrus OCT software.

### Fluorescein Angiography

Fluorescein angiography (FA) was performed on anesthetized animals, using an Optos 200Tx ultra-wide field retinal imaging system (Optos PLC, Dunfermline, Scotland, UK), 14 days following laser photocoagulation. Photographic images were captured after 0.4 mL 10% fluorescein sodium (Alcon Laboratories, Inc., Fort Worth, TX) was injected into the peritoneum. Two masked retinal specialists, not involved in laser photocoagulation or angiography, evaluated FA images. Lesions were graded using a previously established grading system [24]. Grade 0 was given if no hyperfluorescence was present in the early and late phases. Grade 1 was given if hyperfluorescence without leakage was present in the early or late phase. Grade 2A was given if hyperfluorescence in the early or middle phase and leakage in the late phase was observed. With this grade, late leakage did not extend beyond the treated areas. Grade 2B was given if bright hyperfluorescence in the middle phase and leakage beyond treated areas in the late phase existed. Grade 2B lesions were regarded as clinically significant [24]. Grade 0 lesions were excluded from analyses because the laser injury may not have been sufficient to induce CNV.

### Flat Mount Staining and Choroidal Neovascularization Size Measurement

The size of CNV lesions was measured in RPE-choroid-sclera flat mounts. Fourteen days following laser photocoagulation, rats were anesthetized and perfused with 5 mL phosphate-buffered saline containing 50 mg/mL of fluorescein isothiocyanate (FITC)-labeled dextran (2×10^6^ average molecular weight, Sigma, St. Louis, MO). Eyes were then enucleated and fixed for 1 hour in 4% phosphate-buffered paraformaldehyde. The anterior segment was removed and the entire retina was carefully dissected from the eye cup. The RPE/choroid complex was flattened by making four radial incisions with the sclera facing down. Flat mounts were examined under a laser confocal microscope (LSM510 META, Carl Zeiss, Jena, Germany) and images of laser-induced lesions were captured. The CNV lesions were identified as fluorescent blood vessels on the choroidal/retinal interface surrounded by a region with no fluorescence. An operator masked to treatment group assignment used images to measure CNV area in each laser burn using Carl Zeiss LSM Image Browser software (version 4.0.0.241). Meanwhile, lesion quantification was also performed in confocal images of RPE-choroid-sclera flat mounts labeled with tetramethylrhodamine isothiocyanate (TRITC)-conjugated isolectin B4 (Sigma-Aldrich, Gillingham, UK). The 3D structure of the CNV complex was constructed from a z-series of CLS images using IMARIS software 7.6.5 (Bitplane, Zürich, Switzerland).

### Histological Analysis of Retinal-Retinal Pigment Epithelium-Choroid Cross Sections

Paraffin-embedded tissue slices, through lesions, were examined using hematoxylin and eosin (H&E) staining. Fourteen days after CNV induction, eyes were enucleated and immediately immersed in a mixture of 2.5% glutaraldehyde and 4% paraformaldehyde for 24 hours. Tissue samples were dehydrated and embedded in paraffin. Serial sections, 4-µm thick, were then cut with a microtome and examined to determine the center of each lesion. Sections were stained with H&E for light microscopy, during which an operator masked to treatment assignment measured CNV lesion dimensions on digitized histological images using the Axiovision 4.8.2 software (Carl Zeiss MicroImaging GmbH). Maximum CNV thickness (*X*), measured from the bottom of the pigmented choroidal layer to the top of the neovascular membrane, was determined. The thickness of the intact pigmented choroid adjacent to the lesion (*Y*) was also measured and the CNV/choroidal thickness ratio (*X/Y*) was calculated.

### Quantification of VEGF, MMP-2, and MMP-9 by Enzyme-Linked Immunosorbent Assay

Animals were also administered a daily dose of their assigned oral drug beginning 3 days before laser photocoagulation and ending 7 days after laser photocoagulation. After Day 7, animals were sacrificed and each whole retina was dissected from the RPE-choroid complex. Proteins from these two tissues were then isolated separately. Samples were sonicated on ice in lysis buffer (20 mM imidazole HCl, 10 mM KCl, 1 mM MgCl_2_, 10 mM EGTA, 1% Triton X-100, 10 mM NaF, 1 mM Na molybdate, and 1 mM EDTA with protease inhibitor; Sigma-Aldrich). The resulting lysate was centrifuged at 13 000 rpm for 5 min at 4°C, and some supernatant was transferred to a fresh tube. Supernatant VEGF levels were then quantified with an enzyme-linked immunosorbent assay (ELISA) kit specific for VEGF-A (rat VEGF immunoassay kit, detection threshold = 2 pg/mL, ab100787, Abcam, Cambridge, MA). Some supernatant was also transferred to a fresh tube to quantify MMP-2 and MMP-9 levels with ELISA kits (rat MMP-2 immunoassay kit, detection threshold = 10 pg/mL, IBL-America, Minneapolis, MN; rat MMP-9 immunoassay kit, detection threshold = 1.1 ng/mL; Uscn Life Science, Wuhan, China). All readings were taken using a microplate spectrophotometer system (Spectramax 190, Molecular Devices, Sunnyvale, CA) at 450 nm and were normalized for total protein content. Choroid and sclera were prepared in the same way as retina for measuring VEGF, MMP-2, and MMP-9 levels. All assay measurements were performed in triplicate.

### Cell cultures

The ARPE-19 cells were purchased from American Type Culture Collection (ATCC; Manassas, VA) and were used for human RPE cells. The cells were routinely maintained in Dulbecco’s modified Eagle’s medium/F-12 (DMEM/F-12; Hyclone, Logan, UT) containing 10% fetal bovine serum, 100 U/mL penicillin, and 100 µg/mL streptomycin. Experiments were performed using cells between passages 22 and 25.

Human umbilical vein endothelial cells (HUVECs) were purchased from ATCC and were grown in a gelatin-coated dish in M199 medium supplemented with 20% fetal bovine serum, 100 U/mL penicillin, 100 µg/mL streptomycin, 3 ng/mL basic fibroblast growth factor, and 1 mL of heparin (all reagents from Gibco BRL, Carlsbad, CA). HUVECs used in this study were taken from passage 4.

### Measurement of VEGF secretion from ARPE-19 monolayers

Approximately 100 000 cells/cm^2^ were seeded and cultured for 48 hours to reach confluent monolayers. After being rinsed with phosphate buffered saline, cells were withdrawn from serum for 24 hours. Cells were incubated with treatment of 150 µM tertiary-butylhydroperoxide (t-BH; Sigma) with or without 25 µg/mL of *M. officinalis* extract for 5 hours. At the end of the experiment, the medium was collected and analyzed by ELISA kit procured from Invitrogen (Carlsbad, CA) to measure the levels of VEGF.

### Total RNA extraction and real time-polymerase chain reaction (PCR)

ARPE-19 cells were treated with 150 µM t-BH with or without 25 µg/mL of *M. officinalis* extract for 5 hours. HUVECs were treated with 100 µM t-BH with or without 25 µg/mL of *M. officinalis* extract for 2 hours. Total RNA was extracted from freshly removed ARPE-19 cells and HUVECs using RNeasy Mini kit (Qiagen, Valencia, CA) according to the manufactures protocol. Then, cDNA was synthesized from total RNA using a reverse transcription reaction (EcoDry cDNA Synthesis Premix; Takara Bio, Shiga, Japan). The cDNA was amplified in triplicate using real-time PCR with the ABI Prism 7700 Sequence Detection System (Applied Biosystems, Lincoln, CA) using optical grade 96-well plates. The PCR-primers and TaqMan probes for MMP-2, MMP-9, and VEGF were purchased from Applied Biosystems (Assay ID: Hs01548727_m1, Hs00234579_m1, and Hs00900055_m1, respectively; Applied Biosystems, Darmstadt, Germany). Glyceraldehyde phosphate dehydrogenase (GAPDH), a constitutively expressed housekeeping gene, was also amplified under the same conditions and used to normalize reactions. The following PCR conditions were used. After initial activation of uracil-*N*-glycosylase at 50°C for 2 min, AmpliTaq Gold was activated at 95°C for 10 min. PCR consisted of 45 amplification cycles (denaturation at 95°C for 15 s, annealing at 60°C for 1 min, and extension at 60°C for 1 min). During PCR amplification, the amplified product amount was monitored by continuous measurement of fluorescence. The expression level of target gene was normalized to internal GAPDH and represented as relative expression.

### Statistical Analysis

Data are expressed as mean ± standard deviation of the mean, where applicable. Statistical analyses were performed using the Kruskal-Wallis test for comparison between several groups and the Mann-Whitney *U* test for comparison between the two subgroups to assess the effects of drug treatment. Statistical significance was defined as *P*<0.05. All statistical analyses were performed using SPSS software for Windows (version 21.0, SPSS, Inc., Chicago, IL).

## Results

### Effect of *M. officinalis* Extract on CNV Thickness; Quantification of CNV using SD-OCT

**Figure 1:**
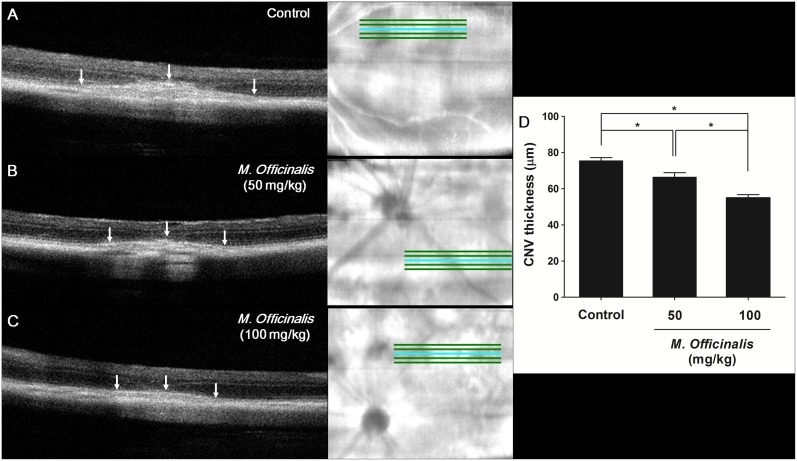
Spectral-domain optical coherence tomography images of CNV. (A) Control group. (B) Low-dose *M. officinalis* extract (50 mg/kg) group. (C) High-dose *M. officinalis* extract (100 mg/kg) group. White arrows indicate a spindle-shaped choroidal neovascularization (CNV) lesion. (D) Measurement of CNV thickness, *in vivo*, showed significantly smaller CNV in *M. officinalis* extract-treated rats than in control rats. The response was dose dependent. Data are presented as mean ± standard deviation. *indicates statistical significance (*P*<0.05).

The effect of *M. officinalis* extract on CNV lesion thickness was examined using SD-OCT images obtained 14 days after laser photocoagulation. Mean CNV thickness was significantly lower in both the low-dose (66.33±8.11 µm, *P* = 0.023) and high-dose (54.95±5.23 µm, *P*<0.001) treatment groups than in the control group (75.38±6.39 µm, Figure 1). The difference in CNV thickness between the low-dose and high-dose treatment groups was also statistically significant (*P* = 0.003).

### Inhibitory Effect of *M. officinalis* Extract on CNV Size; Dimensions of CNV in Choroidal Flat Mounts and Histology

**Figure 2:**
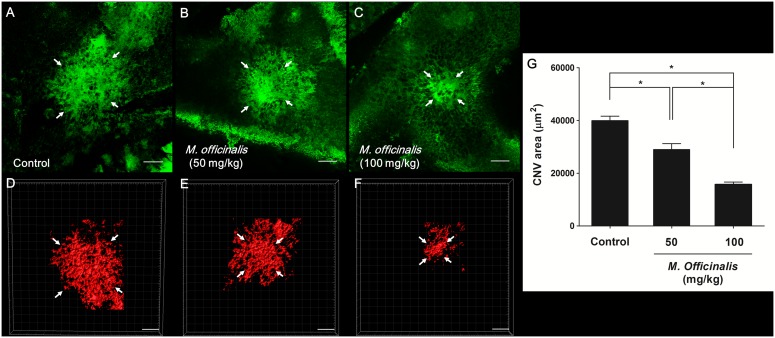
Fluorescent images of CNV in retinal pigment epithelium-choroid-sclera flat mounts. (A, D) Control group. (B, E) Low-dose *M. officinalis* extract (50 mg/kg) group. (C, F) High-dose *M. officinalis* extract (100 mg/kg) group. (A, B, C) Representative images of fluorescein isothiocyanate (FITC)-dextran staining (*green*). (D, E, F) Representative images of tetramethylrhodamine isothiocyanate (TRITC)-conjugated isolectin B4 staining (*red*). White arrows indicate laser-induced choroidal neovascularization (CNV) size. The scale bar represents 100 µm. (D) Mean CNV area was significantly lower in *M. officinalis* extract-treated rats than in control rats. The response was dose dependent. Data are presented as mean ± standard deviation. *indicates statistical significance (*P*<0.05).

**Figure 3:**
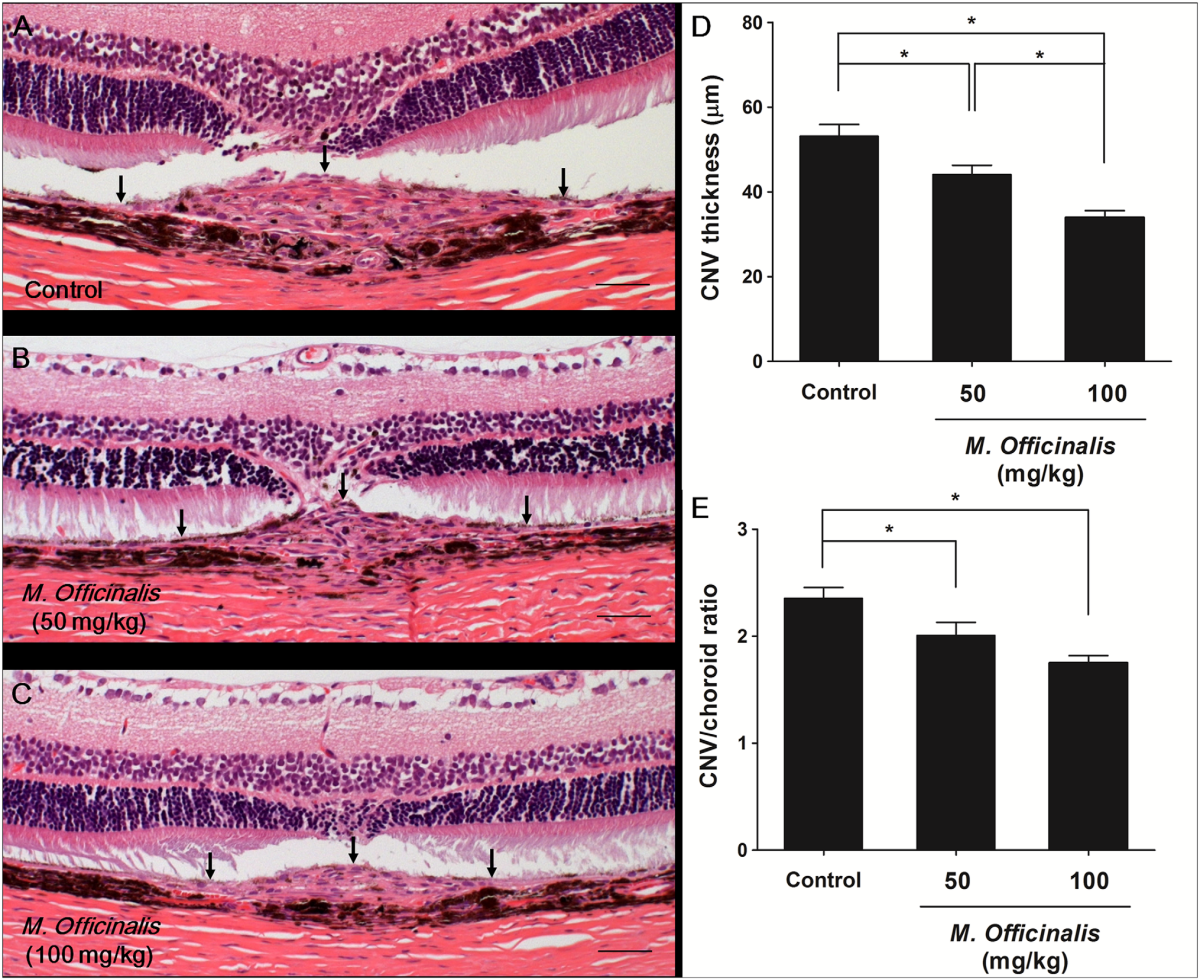
Hematoxylin and eosin staining of paraffin-embedded rat eye sections through areas of CNV. (A) Control group. (B) Low-dose *M. officinalis* extract (50 mg/kg) group. (C) High-dose *M. officinalis* extract (100 mg/kg) group. Black arrows indicate choroidal neovascularization (CNV) lesion margins. Scale bar indicates 50 µm. (D) Rats treated with *M. officinalis* extract had significantly smaller CNV lesion than controls. The response was dose dependent. (E) Mean CNV/choroid thickness ratios were significantly lower in *M. officinalis* extract-treated rats than in control rats. Data are presented as mean ± standard deviation. *indicates statistical significance (*P*<0.05).

The size of CNV lesions was examined using choroidal flat mounts and histology and was examined 14 days after laser photocoagulation. Examination of choroidal flat mounts revealed that mean CNV area was significantly lower in both the low-dose (29 115±12 766 µm, *P*<0.001) and high-dose (15 760±5 642 µm, *P*<0.001) treatment groups than in the control group (39 931±9 529 µm, Figure 2). Histological analysis showed that the mean value of the largest CNV lesion diameter was smaller in the low-dose (44.09±12.01 µm, *P* = 0.016) and high-dose (33.93±7.64 µm, *P*<0.001) treatment groups than in the control group (51.00±12.37 µm) (Figure 3, A–D). The mean CNV/choroid ratio was also significantly lower in the low-dose (2.01±0.66, *P* = 0.018) and high-dose (1.75±0.32, *P*<0.001) treatment groups than in the control group (2.36±0.60). The CNV/choroid thickness ratio was not significantly different between the low-dose and high-dose treatment groups (*P* = 0.165) (Figure 3, E).

### Anti-Angiogenic Effect of *M. officinalis* Extract on CNV; Angiographic Leakage from CNV

**Figure 4:**
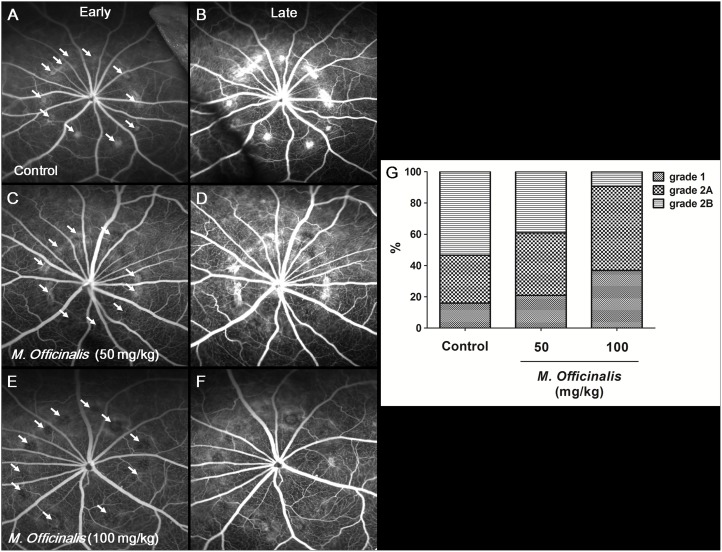
Fluorescein angiographic images. Early (A, C, E) and late (B, D, F) phase fluorescein angiographic images taken 14 days after laser injury to induce choroidal neovascularization (CNV). (A, B) Control group. (C, D) Low-dose *M. officinalis* extract (50 mg/kg) group. (E, F) High-dose *M. officinalis* extract (100 mg/kg) group. White arrows indicate laser treatment location. (G) Histogram of angiographic leakage grades. Significantly fewer grade 2B lesions were observed in the high-dose group than in the control group.

To evaluate the *in vivo* effect of *M. officinalis* extract on angiogenesis, FA was performed 14 days after laser photocoagulation. Comparison of angiograms between groups revealed that both the low-dose and high-dose treatment groups developed CNV lesions that were less leaky than those in the control group. Figure 4 illustrates the proportion of CNV lesions with fluorescein leakage in each group. Pathologically significant leakage (grade 2B lesions) was observed in 53.4%, 39.1%, and 9.2% of lesions in the control, low-dose, and high-dose groups, respectively. The difference in proportion of grade 2B lesions between the high-dose and control groups (*P*<0.001), and the high-dose and low-dose groups (*P*<0.001) were statistically significant.

### Down-Regulation of VEGF, MMP-2, and MMP-9 by *M. officinalis* Extract

**Figure 5:**
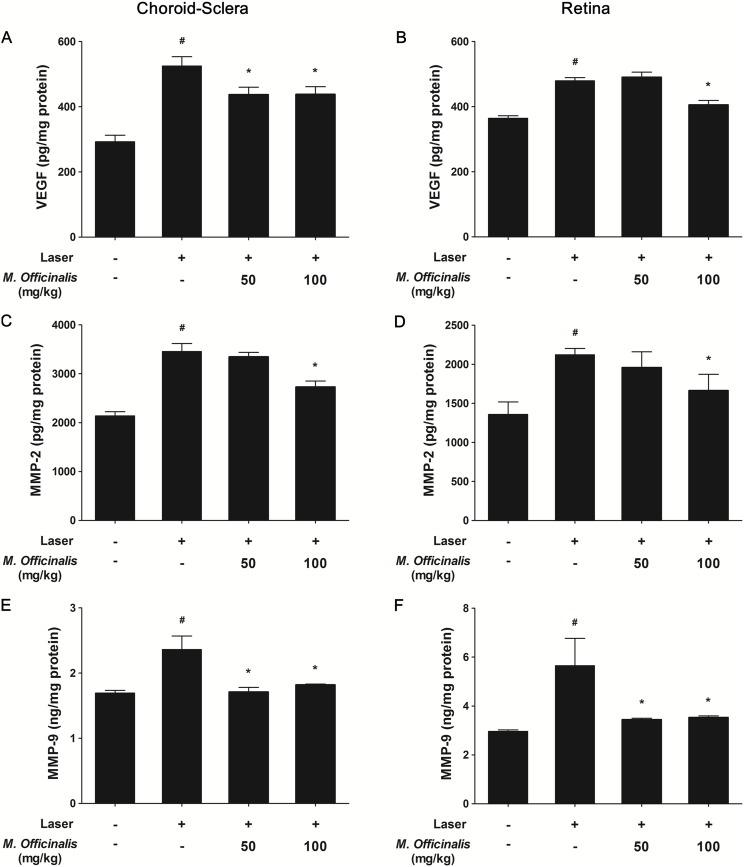
Quantification of VEGF, MMP-2, and MMP-9 levels in choroidal-scleral complex and retina. Levels of vascular endothelial growth factor (VEGF), matrix metalloproteinase (MMP)-2, and MMP-9 were evaluated in the choroidal-scleral complex (A, C, E) and retina (B, D, F). (A, B) Mean normalized VEGF levels in choroidal-scleral and retinal tissues were significantly lower in high-dose *M. officinalis* extract (100 mg/kg) group than in controls. The low-dose *M. officinalis* extract (50 mg/kg) group only had significantly lower VEGF levels than controls in choroidal-scleral tissues. (C, D) Mean normalized MMP-2 levels in choroidal-scleral and retinal tissues were significantly lower in high-dose extract rats than in controls. (E, F) Mean normalized MMP-9 levels in choroidal-scleral and retinal tissues were significantly lower than controls in both high- and low-dose groups. Data are presented as mean ± standard deviation. #, *indicate significant difference (*P*<0.05) in comparison to negative control or the vehicle-treated group, respectively.

To determine whether anti-angiogenic properties of *M. officinalis* extract on CNV was mediated by anti-VEGF and MMP-inhibitory activities, the VEGF, MMP-2, and MMP-9 levels of the choroid-sclera and retina were measured 7 days after CNV induction. As shown in Figure 5A and 5B, mean choroidal-scleral (438.03±40.38 pg/mg) and retinal (405.30±27.10 pg/mg) VEGF levels were significantly lower in the high-dose treatment group than in the control group (choroid-sclera: 525.07±49.09 pg/mg, *P* = 0.029; retina: 479.30±19.43 pg/mg, *P* = 0.029). The VEGF levels were significantly lower in the low-dose treatment group, than in the control group in choroidal-scleral tissue (437.13±39.61 pg/mg, *P* = 0.029), but not in retinal tissue (490.38±31.26 pg/mg, *P* = 0.686). Mean normalized MMP-2 level in the high-dose group was 2 729.16±211.45 pg/mg in choroidal-scleral tissue and 1 662.87±361.90 pg/mg in retinal tissue, both of which were significantly lower than in the control group (choroid-sclera: 3 454.15±281.66 pg/mg, *P* = 0.039; retina: 2 121.56±140.21 pg/mg, *P* = 0.049). However, differences in MMP-2 levels between the low-dose (choroid-sclera 3 345.49±160.73 pg/mg, *P* = 0.509; retina: 1 958.61±346.73 pg/mg, *P* = 0.309) and control groups were not statistically significant (Figure 5C and 5D). Mean MMP-9 levels were significantly higher in controls than in the high-dose and low-dose treatment groups. Choroidal-scleral MMP-9 level was 2.36±0.42 ng/mg, 1.71±0.14 ng/mg (*P* = 0.029), and 1.82±0.02 ng/mg (*P* = 0.038) in the control, low-dose, and high-dose groups, respectively. Retinal MMP-9 level was 5.65±2.24 ng/mg, 3.44±0.11 ng/mg (*P* = 0.019), and 3.53±0.13 ng/mg (*P* = 0.019) in the control, low-dose, and high-dose groups, respectively (Figure 5E and 5F).

### Inhibition of t-BH-induced VEGF Expression by *M. officinalis* Extract; ELISA

**Figure 6:**
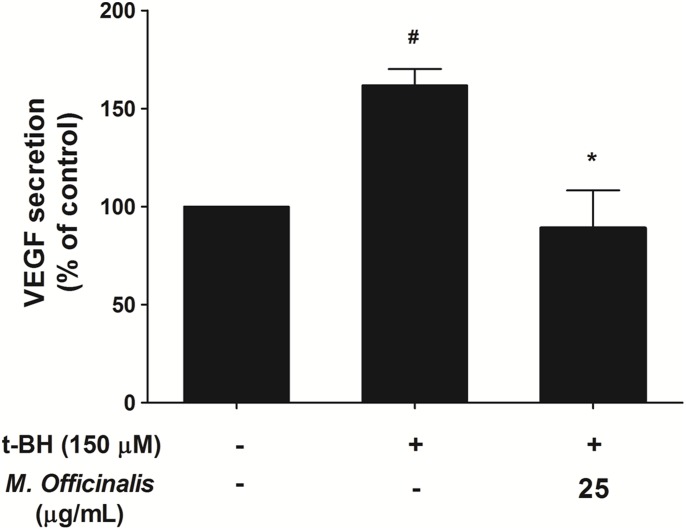
Quantification of VEGF secretion after treatment of t-BH on ARPE-19 cells. Mean normalized tertiary-butylhydroperoxide (t-BH)-induced vascular endothelial growth factor (VEGF) expression was significantly lower with cotreatment of 25 µg/mL of *M. officinalis* extract. Data are presented as mean ± standard deviation. #, *indicate significant difference (*P*<0.05) in comparison to negative control or the t-BH alone group, respectively.

To evaluate whether anti-angiogenic effect of *M. officinalis* extract was mediated by anti-oxidant activity of *Melissa* extract, we measured VEGF expression after treatment of t-BH on ARPE-19 cells. As shown in Figure 6, t-BH-induced VEGF expression (1.6-fold, *P* = 0.018) was significantly reduced to 0.9-fold compared with control with cotreatment of 25 µg/mL of *M. officinalis* extract (*P* = 0.040).

### Effect of *M. officinalis* Extract on t-BH-induced VEGF, MMP-2, and MMP-9 Transcription in ARPE-19 cells and HUVECs; Real-time quantitative PCR

**Figure 7:**
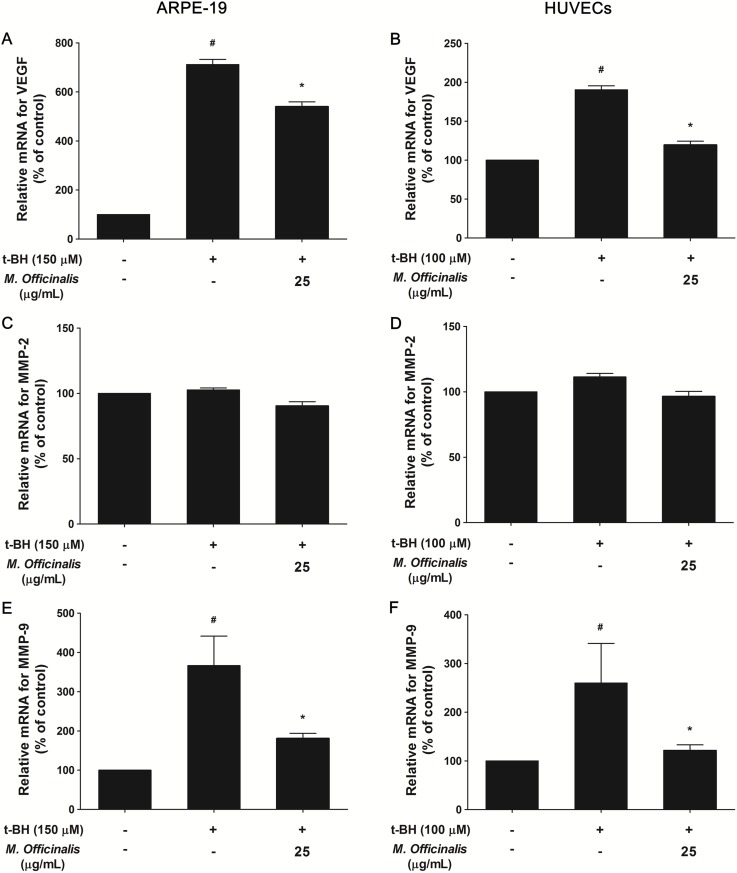
VEGF, MMP-2, and MMP-9 expression after treatment of t-BH on ARPE-19 cells and HUVECs. Expression of mRNAs for vascular endothelial growth factor (VEGF), matrix metalloproteinase (MMP)-2, and MMP-9 determined by real-time quantitative polymerase chain reaction (PCR) were evaluated in human retinal pigment epithelial (ARPE-19) cells (A, C, E) and in human umbilical vein endothelial cells (HUVECs) (B, D, F). (A, B) The relative VEGF mRNA levels, which are induced by tertiary-butylhydroperoxide (t-BH), were reduced significantly with cotreatment of 25 µg/mL of *M. officinalis* extract. (C, D) The expression of MMP-2 mRNA did not change in the t-BH treated cells, nor did that in the t-BH and *M. officinalis* extract treated cells. (E, F) The relative MMP-9 mRNA levels decreased significantly with cotreatment of 25 µg/mL of *M. officinalis* extract. Data are presented as mean ± standard deviation. #, *indicate significant difference (*P*<0.05) in comparison to negative control or the t-BH alone group, respectively.

The expression levels of VEGF, MMP-2, and MMP-9 mRNA were measured by quantitative real-time PCR after t-BH treatment in the ARPE-19 cells and HUVECs (Figure 7). In ARPE-19 cells, the t-BH-induced VEGF expression (7.1-fold, *P*<0.001) decreased 5.4-fold compared to the control with cotreatment of 25 µg/mL of *M. officinalis* extract (*P* = 0.039). The expression of MMP-2 mRNA did not change in the t-BH treated cells, nor did that in the t-BH and *M. officinalis* extract treated cells. Notably, the t-BH-induced MMP-9 expression (3.7-fold, *P* = 0.020) decreased significantly after cotreatment of 25 µg/mL of *M. officinalis* extract (1.8-fold, *P* = 0.035). In HUVEC, t-BH-induced VEGF (1.9-fold, *P* = 0.003) and MMP-9 (2.6-fold, *P* = 0.040) transcription were significantly reduced to 1.2-fold (*P* = 0.001) and 1.2-fold (*P* = 0.035), respectively, compared with control with cotreatment of 25 µg/mL of *M. officinalis* extract. However, the expression of MMP-2 mRNA did not change in the t-BH treated cells, nor did that in the t-BH and *M. officinalis* extract treated cells.

## Discussion

*Melissa officinalis* belongs to the Lamiaceae family, is a perennial herb which has been used in folk medicine for many years. In addition to antioxidant [25], [26], sedative [27], anti-inflammatory, hepatoprotective, digestive, antibacterial, antifungal, antiviral [20], [21], [28], antihistaminic, antikinetic, antilipidaemic [29], anxiolytic [30], and protective activity against Alzheimer disease [25], [31], *M. officinalis* was recently reported to have anti-angiogenic activity [22], [23] in obese mice. Phytochemical studies carried out with *M. officinalis* have demonstrated the presence of numerous constituents, including polyphenolic compounds (rosmarinic acid, caffeic acid and protocatechuic acid), essential oils, monotherpenoid aldehides, sesquiterpenes, flavonoids (luteolin) and tannins [19], [27], [29], [32]. Taking into account the biological activities of *M. officinalis*, it is surprising that no pharmacological study has been carried out on the possible anti-angiogenic effects of the extract on CNV up to now.

In the present study, we demonstrated for the first time that *M. officinalis* extract inhibits experimental CNV in a dose-dependent manner. Both CNV area and thickness was significantly reduced in extract-treated rats compared with vehicle-treated rats. The percentage of eyes with significant fluorescein leakage was also significantly lower in extract-treated rats than in control rats. Furthermore, expression levels of VEGF, MMP-2, and MMP-9 in the choroidal-scleral complex and the retina were lowered by the oral administration of *M. officinalis* extract. However, MMP-2 mRNA level did not change in the t-BH and *M. officinalis* extract treated APRE-19 cells and HUVECs, while there was an inhibition of MMP-2 upon treatment with *M. officinalis* extract *in vivo*. The discrepancy between the *in vivo* and *in vitro* results may be explained in several ways. First, t-BH may possibly not be the appropriate substance for inducing MMP-2 expression in these cells, even though t-BH was used because oxidative stress played a crucial role in the development of laser-induced CNV and t-BH is a suitable substance to evaluate the anti-oxidative activity of *M. officinalis* extract in an *in vitro* setting. Nevertheless, our results that only minimal induction of MMP-2 mRNA occurred in these cells under the oxidative stress are consistent with previous reports by others [33], [34]. Second, MMP-2 induction in an *in vivo* laser-induced CNV model could possibly be attributed to other miscellaneous causes besides oxidative stress during CNV formation. Third, MMP-2 could be derived from other cell types besides RPE and endothelial cells. Taken together, our results suggest that the CNV inhibitory effect of *M. officinalis* extract is primarily via VEGF and MMP-9 down-regulation.

Oxidative stress and the production of ROS seem to play a critical role in AMD pathogenesis [35]. Even though exact pathways for the anti-angiogenic effect of *M. officinalis* extract down-regulating VEGF and MMPs are still being investigated, results from the previous studies provide some clues. Rosmarinic acid, the major compound of the *M. officinalis* extract, was reported to have the anti-angiogenic activity to intrinsic angiogenic phenotypes of HUVECs without exogenous VEGF stimulation and suppress ROS-mediated VEGF expression and IL-8 release [36]. It also suppresses retinal neovascularization in a mouse model of retinopathy of prematurity via G_2_/M phase cell cycle arrest with increase of p21^WAF1^ expression [37]. Moreover, caffeic acid, another major compound of the *M. officinalis* extract, was reported to effectively suppress pathological angiogenesis in tumor through inhibiting the activity of STAT3-HIF1α [38]. It also was found to inhibit VEGF-induced proliferation, migration of retinal endothelial cells, and *in vitro* angiogenesis of tube formation by suppression of the H_2_O_2_-induced ROS production and VEGF expression [39]. While the mechanism of *M. officinalis* extract *in vivo* is not completely clear, our results suggest that the anti-angiogenic potential of *M. officinalis* extract might be related to anti-oxidative activity of its active components, rosmarinic acid and caffeic acid, which further resulted in the inhibition of ROS-associated VEGF and MMPs expression. While this is encouraging, additional research is necessary to further reveal the exact mechanism by which *M. officinalis* extract inhibits CNV development.

Using an herbal medicine as a monotherapy for exudative AMD is highly controversial considering the potential placebo effect. Furthermore, producing standardized herbal formulas with consistent active ingredient levels is still challenging. Above all, a laser-induced CNV rat model does not perfectly mimic exudative AMD in humans. It is a model of acute injury and inflammation, rather than one of long standing senile degeneration and chronic inflammation [40]. Nevertheless, there are several reasons why the effect of *M. officinalis* extract on CNV deserves attention. First, *M. officinalis* extract can be administered systemically via the oral route, and *Melissa* extract has a good safety profile with its use in traditional herbal medicine. Current therapeutic strategies for exudative AMD involve multiple intravitreal injections of anti-VEGF agents. Even though these injections have been well-tolerated in human eyes and these agents have minimal ocular and systemic toxic potential, repeated intravitreal injections carry the risk of serious ocular adverse events such as endophthalmitis [41]. Therefore, an oral anti-CNV drug with a good safety and tolerability profile could provide an alternative, effective approach for wet AMD therapy. In addition, *M. officinalis* extract and anti-VEGF treatments could have synergistic properties and, together, have the potential to reduce the economic burdens and clinical risks related to repeated intravitreal anti-VEGF agent injections. The concomitant therapy with intravitreal anti-VEGF and systemic *M. officinalis* extract would merit further human studies.

In conclusion, our study shows that orally administered *M. officinalis* extract significantly inhibits CNV development in rats, which might be associated with its anti-oxidative activity via inhibition of VEGF and MMP-9 expression. We suggest that *M. officinalis* extract is a potential candidate drug as an adjuvant therapy for treating exudative AMD.
